# Our Contemporaries

**Published:** 1916-01

**Authors:** 


### OUR CONTEMPORARIES.

---

We refer to the Obituary Dept. for a notice of Dr. Kenneth W. Millican, well known as an editor in this country and England.

---

The Medical Review of Reviews has been purchased by Dr. Wm. J. Robinson, editor of the Critic and Guide and the

American Urologic Review. The former editor, for four years, was Dr. Ira S. Wile.

